# The influence of *Cistus incanus* L. leaves on wheat pasta quality

**DOI:** 10.1007/s13197-019-03900-9

**Published:** 2019-07-11

**Authors:** Katarzyna Lisiecka, Agnieszka Wójtowicz, Dariusz Dziki, Urszula Gawlik-Dziki

**Affiliations:** 1 Department of Thermal Technology and Food Process Engineering, University of Science in Lublin, Głęboka 31, 20-612 Lublin, Poland; 2 Department of Biochemistry and Food Chemistry, University of Life Sciences in Lublin, Skromna 8, 20-704 Lublin, Poland

**Keywords:** Pasta, *Cistus incanus*, Extraction method, Antioxidant activity, Cooking quality

## Abstract

Modern nutritional trends and looking for functional food and dedicated products for various consumers are a source of inspiration for scientists to develop new pro-health supplemented foods with high quality. Therefore, the present study aimed to determine the selected properties of common wheat pasta fortified with dried *Cistus incanus* in amount from 1 to 5% as a replacement of wheat flour. Pasta was made with a spaghetti shape and dried. Supplemented pasta was tested for total phenolics content, the total activity against DPPH, the ability to neutralize free radicals to ABTS and the antioxidant capacity reduction power, using various extraction procedures. Selected physicochemical properties of pasta were evaluated: cooking time, cooking weight, cooking loss, hardness and color profile of dry and cooked pasta in CIE-Lab scale, as well as the sensory properties of supplemented products. The addition of *C. incanus* to fortify wheat pasta increased total phenolics content and antioxidant activity with some significant differences according to the extraction procedure used. Methanolic extraction was more efficient than buffer extraction. Increased addition of dry *Cistus* herb caused higher cooking weight, cooking loss and increased hardness of cooked pasta. Studies have shown that *C. incanus* addition had a slight effect on color change with the largest decrease in brightness, especially for cooked products. Finally, it was found that advisable application of *C. incanus* addition to achieve nutritionally improved composition of pasta should not exceed 3% due to the proper sensory characteristics.

## Introduction

New nutritional trends, as well as increased consumer awareness, caused the introduction into food health-promoting additives, especially into cereal products (Oniszczuk et al. 2016a, b). However, wide range of conventional products, from bread to baked products, extrudates, pasta and cereal-based foods are suitable for enrichment with functional additives as rich in e.g. fiber, polyphenols, as well as with increased antioxidant activity in order to achieve functional products (Biernacka et al. 2017; Bouasla et al. 2016; Oniszczuk et al. 2015a; Wójtowicz et al. 2018).

The main ingredient in traditional pasta processing is durum wheat, but also there are several pasta assortments made with common wheat or other raw materials. According to the low cost, simple production process and specific sensory attributes, pasta is a good example of versatility product suitable for the nutritional fortification (Sant’Anna et al. 2014). Wheat pasta enriched i.e. with herbs like oregano leaves (Boroski et al. 2011), vegetables like carrot pomace, spinach, tomatoes (Gull et al. 2015; Rekha et al. 2013), legumes like vicia faba bean, yellow pea, lentil, white bean, soy proteins (Wójtowicz and Mościcki 2014), pseudocereals like amaranth (Cárdenas-Hernández et al. 2016), specific plants like wakame, carob or chia (Sęczyk et al. 2016) or by-products like bran or grape marc (Sant’Anna et al. 2014) are the examples of designedly supplementation with bioactive ingredients.

*Cistus incanus* L. is a herb plant that is rich in pro-health compounds. There is mentioned in the literature that herbal infusions and teas from *C. incanus* contain various polyphenolic compounds, in particular phenolic acids, flavonoids, especially flavone-3-ol derivatives as well as essential oils and resins (Viapiana et al. 2017). In the literature *C. incanus* is known also as *Cistus creticus* (Demetzos et al. 1997). *Cistus creticus* L. can be found in the Mediterranean and the Black Sea regions. The raw material is dried leaves which contain phenolic acids (gallic acid, ellagic acid, *p*-coumaric acid), flavonoids (quercetin, hyperoside, rutin), phytosterols, mucilages and tannins (Santagati et al. 2008). Essential oils found in *C. creticus* subsp. *eriocephalus* contain several active components as manyol-oxide (manjol), α-kadinen, δ-kadinen, bulnezol, wiridiflorol, ledol, α-kopaen, β-selinen, kubenen and 13-epi-manyol oxide, and showed antibacterial properties (Demetzos et al. 1997). On the global market *C. incanus* is spread as dry herbs, teas and dietary supplements. In the literature it can be found information on tests performed on commercial teas, which confirm the presence of pro-health compounds and some benefits as the prevention of caries and periodontitis. It was confirmed also the antibacterial activity of *C. incanus* herbal tea on *Streptococcus mutans* (Viapiana et al. 2017). Móricz et al. (2018) used TLC-DB to obtain antibacterial profile of eleven *C. incanus* herbal teas and to guide isolation of bioactive compounds, rich exclusively in flavonoid aglycons, from the appropriate TLC zones. However, for *C. incanus* extracts it was not completed the evaluation of antibacterial and antifungal activity yet. But there are any information about food supplementation with this herb.

The aim of the study was to determine the effect of the addition of dried leaves of *C. incanus* on the total polyphenols content and antioxidant activity of health-promoting compounds with application of various extraction methods, as well the effect of additive on selected properties of pasta products as the color profile, culinary quality and sensory characteristics of supplemented common wheat pasta.

## Materials and methods

### Plant materials and pasta processing

For pasta preparation wheat flour type 500 (PZZ Lubella Sp. z o. o. Sp. k., Lublin, Poland) was used as a basic raw material and water was added in ratio of 5:1. The chemical composition of wheat flour per 100 g was following: energy value 1485 kJ/350 kcal, fat 1.4 g (including saturated fatty acids 0.4 g), carbohydrates 73.0 g (including sugars 2.3 g), fiber 2.8 g, protein 10.0 g, salt 0.03 g (producer data). As an additive the dried leaves of *C. incanus* herb were purchased (originated from Albania, distributor Malwa, Lubiszyn, Poland) and ground with a knife mill (Grindomix GM200, Retsch, Germany) for granulation of < 200 μm. The chemical composition of *C. incanus* per 100 g, tested according to AACC methods (2011) was as follows: fat 1.36 g, protein 7.88 g, ash 7.70 g, total fiber 63.74 g, including 61.78 g of insoluble fiber and 1.96 g of soluble fiber.

Pasta fortified with *C. incanus* was prepared by replacing the wheat flour in the recipe with the addition of dried leaves in amounts of 1, 2, 3, 4 and 5%. Dry ingredients were mixed in a stainless steel bowl with water up to 40% of dough moisture content and shaped for spaghetti with 2 mm forming die using a KitchenAid Artisan7 (Greenville, USA). The produced pasta was dried at 22 °C and 53% humidity for 24 h using Pasta Drying Rack KitchenAid 5KPDR (Greenville, USA) to the final moisture content of 12%. The obtained products were kept closed and used for further research.

### Solvent extraction procedure

The ground pasta (0.5 g, granulation below 300 µm) as well as dry herb of *C. incanus* were extracted separately with 5 mL of 50% methanol (the first type of extract—ME) and 5 mL of phosphate buffered saline (0.01 M phosphate buffer, 0.0027 M potassium chloride, and 0.137 M sodium chloride; the second type of extract—PBSE). Extractants were purchased from Sigma–Aldrich (Poznań, Poland). The obtained extracts were shaken for 30 min using a rotator Multi RS-60 operating at 5000 rpm (Biosan, Riga, Latvia). The homogenate was centrifuged at the same velocity for 10 min at 4 °C using centrifuge MPW-352R (MPW, Warsaw, Poland). The extraction was carried out in double. The supernatants of ME and PBSE extracts were used for further analyses.

### Evaluation of polyphenols content

The total polyphenols content was determined according to Singleton and Rossi (1965). In brief, 0.05 mL of 50% methanol, 0.1 mL of H_2_O and 0.4 mL of Folin reagent (in a ratio of 1:5 with distilled water) were added to 0.05 mL of extracts. After 3 min, 2 mL of 10% sodium carbonate was added to the mixture and vigorously shaken for 1 min. In a reference sample, 0.05 mL of tested extract was replaced by 50% methanol. Subsequently after 30 min in dark place, the absorbance was measured at 712 nm using Microplate Spectrophotometer (Epoch 2, BioTek, USA) and the total phenolics content was expressed by a gallic acid equivalents (GAE) in mg/g of dry weight (d.w.). Measurements were made in triplicate.

### Scavenging ability on 1,1-diphenyl-2-picrylhydrazyl (DPPH) radicals

The scavenging ability on 1,1-diphenyl-2-picrylhydrazyl radical (DPPH) was carried out using the method presented by Harlina et al. (2018). Briefly, the extract (10 µL) was mixed with 250 µL of DPPH ethanol solution (concentration 0.2 mM/L) and next shaken and rested for 15 min. Subsequently, the absorbance was measured at 725 nm using Microplate Spectrophotometer (Epoch 2, BioTek, USA) from 0 to 15 min every 5 min. Antiradical activity was expressed by EC_50_ (mg d.w./mL) as an effective concentration at which 50% of radicals were scavenged. For each sample and extract type measurements were made in triplicate.

### Scavenging ability on 2,2′-azino-bis(3-ethylbenzthiazoline-6-sulphonic acid) (ABTS) radicals

The ability to neutralize free radicals against ABTS was carried out according to the methodology presented by Re et al. (1999). 10 μL of the sample was added to 250 μL of the ABTS radical. Then, the decrease in absorbance at a wavelength of 734 nm after 2 min was measured using microplate spectrophotometer (Epoch 2, BioTek, USA). Antiradical activity against ABTS was expressed by EC_50_ (mg/mL) as an effective concentration at which 50% of radicals were scavenged. For each extract type and sample recipe measurements were made triplicate.

### Ferric-reducing antioxidant power (FRAP)

The reducing power was determined according to Oyaizu (1986). 0.5 mL of 200 mM phosphate buffered saline extract and 0.5 mL of a 1% solution of potassium ferricyanide were added to 0.5 mL of the extract. The prepared mixture was insulated at 50 °C for 20 min. Then, 0.5 mL of 10% trichloroacetic acid (TCA) was added and the mixture was rested for few minutes. 1 mL of that mixture was taken and mixed with 1 mL of deionized water and 0.2 mL of 1% iron (II) chloride. Absorption of electromagnetic wave energy was measured at 725 nm using Shimadzu UV-1280 UV–Vis Spectrophotometer. The high absorbance, expressed in nm, was the result of high reduction power. FRAP antioxidant power was expressed by EC_50_ (mg/mL) as an effective concentration at which 50% of radicals were scavenged. For each sample measurements were made in triplicate.

### Cooking quality of pasta

The optimum cooking time (OCT) was established by cooking of 25 g pasta in 300 mL of boiling distilled water in a beaker. During cooking pasta strains were removed from a beaker at 30-s sequence and squeezed between two transparent plastic plates in triplicate. The time when the white centre core disappeared was reported as optimum cooking time (AACC 2011).

The cooking weight index (CWI) of pasta was calculated by dividing the weight of pasta after cooking by the weight of uncooked pasta as a quotient weight of cooked pasta after cooking to optimum cooking time. Measurements were made in triplicate for each sample (Biernacka et al. 2017).

Cooking loss (CL) as the amount of pasta components leached into cooking water was evaluated according to AACC 66-50.01 method (AACC 2011). Cooking loss was measured by evaporating the cooking water after OCT to dryness in an air oven in triplicate.

ZwickRoell BDO-FB0.5TH (Zwick GmbH & Co., Ulm, Germany) universal testing machine was used for evaluation of dry and cooked pasta hardness as the maximum force peak during cutting test. Cutting force (N) was measured with Warner–Bratzler knife double-face truncated at an angle 45° with 3 mm thick and 60 mm long. The head speed during the tests was set at 500 mm/min. A force–time curves were achieved and analysed with *testXpertII*^®^v3.3 from data of 10 replications (Wójtowicz and Mościcki 2014).

### Color profile

Color profile of pasta was measured for dry and cooked pasta using Supercolor HP 2132 colorimeter (Braive Instruments, Oupeye, Belgium) in CIE-Lab scale. The measurements determined chromatic coordinates of L*, a* and b*. A coordinate L* described the brightness in the range from 0 to 100 (black to white). A coordinate a* described the redness-greenness balance in-minus for green color to in-plus for red color. A coordinate b* described the yellowness-blueness balance in-minus for blue to in-plus for yellow (Wójtowicz et al. 2017). For each sample measurements were made in five replications.

### Sensory evaluation

Sensory characteristic of dry and cooked pasta was evaluated according to Bouasla et al. (2016) and Rekha et al. (2013). The assessment was conducted on a 9-point hedonic scale with score 1 for dislike extremely, 5 for nor like nor dislike, to 9 for like extremely, the evolution above 5 was noted as acceptable (Boroski et al. 2011). The evaluation panel consisted fifteen persons (15 males, 30 females, aged 21–50 years) familiar with the definitions of sensory attributes of pasta. The dry and cooked pasta were served in amount of approximately 50 g on white platters. Cooked pasta was tested 5 min after cooking with OCT and rinsing with cold water. The samples were evaluated in a laboratory room with a natural sunlight. The dry pasta samples were assessed for appearance, color and aroma, and the cooked pasta evaluation involved appearance, color, aroma, taste and texture. For all samples the overall acceptability was determined as the average of the points received from the assessment of all the examined features. For dry pasta, as very good appearance was defined pasta with a specific appearance suitable for the type and shape of pasta without cracks or any spots. The perfect color and aroma of pasta should be specific to the raw materials used without foreign flavor. For cooked pasta, very good appearance was defined as smooth surface without sticks and roughness. Color for this type of pasta should be uniform and specific to the raw materials used. Aroma should not contain foreign flavor. Taste should be pleasant and characteristic for the ingredients used. Pasta with good texture should be uniform, soft, firm, compact, not adhesive and not too chewy.

### Statistical analysis

Experimental results were mean ± SD of multiple measurements. Analysis of variance was performed by ANOVA procedures (Statistica 13.1, StatSoft, Poland). Significant differences between means were determined by Tukey test with significance level of α = 0.05. Correlation matrix between the tested features have been prepared and correlations have been identified as significant above 0.600 value at α = 0.05.

## Results and discussion

### Phenolics content in pasta

Polyphenols are the most common antioxidants in a daily diet. Its’ functional effect is attributed to the anti-inflammatory, antibacterial, antiviral and anticarcinogenic activity in human body as well as high antioxidant capacity, and thus beneficial to the human health. Nicoletti et al. (2015) reported the amount of total polyphenol content in various species of *Cistus* plant as GEA equivalent and they found 33.16, 32.51 and 40.51 mg GAE/g in *C. monspeliensis*, *C. villosus* and *C. libanotis*, respectively. The amounts of phenolic compounds of dry *C. incanus* herb methanol extracts (ME) and phosphate buffer saline extracts (PBSE) were found at 301.9 mg GAE/g d.w. and 457 mg GAE/g d.w., respectively. Table 1 shows the content of phenolic compounds in methanol and phosphate buffered saline extracts present in dry pasta. The lowest amount of phenolic compounds (6.64 mg GEA/g d.w.) was found in the ME of wheat pasta without additive. Over twice as more of polyphenols were found in the ME sample supplemented with 5% of *C. incanus*. The increased amount of phenolic compounds in ME varied from 8.45 mg GEA/g d.w. for pasta with 1% of additive up to 15.27 mg GEA/g d.w. for pasta supplemented with 5% of additive. The content of total phenolic compounds found in PBSE samples showed better extractability of these components from dry pasta than in ME (Table 1). The highest amount of phenolic compounds was identified in pasta with 5% of the *C. incanus* content, the phenolic compounds content reached 18.28 mg GEA/g d.w. The least amount of phenolic compounds, 8.71 mg GEA/g d.w., was found in ME of pasta sample without additives. The use of dried *Cistus* plant as an additive in the range up to 5% resulted in an increased phenolics content of about 229.97% if ME was used and 209.87% if PBSE was applied for extraction compared to control sample. The results of phenolic profile and antioxidant capacity of hydromethanolic and aqueous extracts of commercial *C. incanus* products presented by Viapiana et al. (2017) revealed that aqueous extracts of *C. incanus* are richer in phenolic compounds and have stronger antioxidant activities than hydromethanolic extracts. Moreover, they found more effective antibacterial activities of aqueous extracts against Gram-positive than Gram-negative bacteria. Pasta products fortified with 1–5% of carob flour showed the increased amount of phenolic compounds from 5.27 mg/g d.w. for pasta with 1% up to 12.12 mg/g d.w. if 5% of carob flour was applied (Sęczyk et al. 2016). Comparying these data with presented results it can be stated that the addition of *C. incanus* is more beneficial to improve the nutritional quality of wheat pasta than carob flour. Bouasla et al. (2016) reported increased level of total phenolics content in precooked gluten-free rice pasta supplemented with selected legumes: yellow pea, lentil and chick pea, added up to 30%. Chakraborty et al. (2016) noticed that total phenolics content of the pasta and the extruded snacks enriched with a mixture of coriander leaves, curry leaves and fenugreek leaves increased with increrasing the amount of additives. The level of the additives reported in the literature is varying in wide range, but the possibility to apply dried herbs is limited because of its specific intensive aroma and herbal taste. In Table 2 it have been identified significant correlations between phenolics content and antioxidant activity of supplemented pasta. TPC ME was highly positively correlated with the amount of *C. incanus* (0.98) and with TPC PBSE (0.96), and negatively correlated with the DPPH ME and DPPH PBSE (− 0.91 and − 0.94, respectively). On the other hand, TPC PBSE was highly positively correlated with *C. incanus* level (0.99) and negatively correlated with DPPH ME and DPPH PBSE (− 0.98 and − 0.96, respectively), with FRAP ME and FRAP PBSE (− 0.91 and − 0.93, respectively) as well as with aroma of dry pasta (− 0.92).

**Table 1:** The content of polyphenols (mg GAE/g d.w.) in methanol extract (ME) and buffer extract (BE) of dry pasta depend on *Cistus incanus* participation

Addition of *Cistus incanus* (%)	TPC in ME (mg GAE/g)	TPC in BE (mg GAE/g)
0	6.64 ± 0.26^c^	8.71 ± 0.61^d^
1	8.45 ± 0.68^ac^	11.04 ± 0.02^a^
2	10.34 ± 0.22^ab^	12.52 ± 0.28^a^
3	11.13 ± 0.07^ab^	15.73 ± 0.28^b^
4	12.89 ± 0.85^bd^	17.15 ± 0.33^bc^
5	15.27 ± 0.59^d^	18.28 ± 0.13^c^

n = 3; mean ± SD

*TPC* total phenolic content, *ME* methanol extract, *PBSE* phosphate buffered saline extract

^a–d^Means followed by the same letter within a column indicate no significant difference (*p* < 0.05) in Tukey test

**Table 2:**
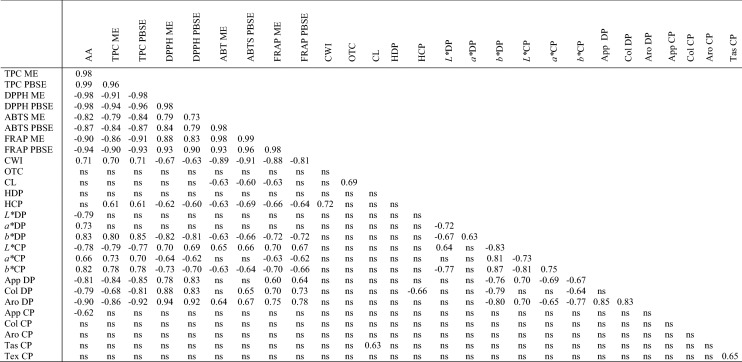
Correlation matrix of selected properties of pasta supplemented with *C. incanus*

Significance level 0.05

*ns* not significant, *AA* additive amount of *C. incanus*, *TPC* total phenolic content, *ME* methanol extract, *PBSE* phosphate buffered saline extract, *DPPH* antiradical activity, *ABTS* antiradical activity, *FRAP* ferric reducing power, *CWI* cooking weight index, *OCT* optimum cooking time, *CL* cooking loss, *H* hardness, *DP* dry pasta, *CP* cooked pasta, L* brightness, a* redness–greenness balance, b* yellowness–blueness balance, *App* appearance, *Col* color, *Aro* aroma, *Tas* taste, *Tex* texture

### Antioxidant activity of pasta tested with DPPH, ABTS and FRAP

**Fig. 1:**
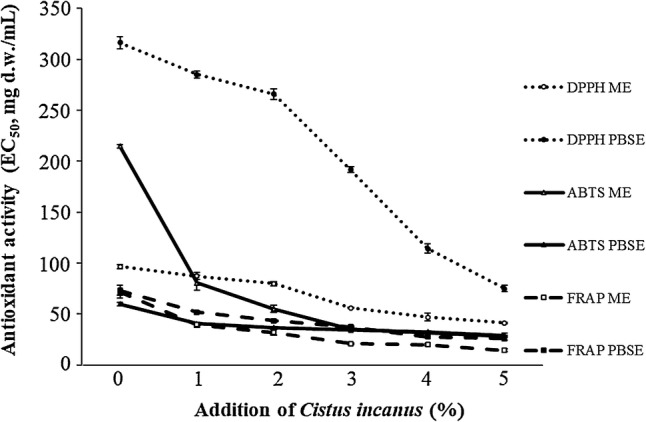
Antioxidant activity of compounds contained in methanol extracts (ME) and phosphate buffered saline extracts (PBSE) of dry wheat pasta depend on *Cistus incanus* participation tested with DPPH, ABTS and FRAP method

DPPH is an example of a long-lived nitrogen radical. Many antioxidants react very slowly on this radical (Gorkem 2016). Determinant of antioxidant activity by DPPH method is the most common method (Boroski et al. 2011). The scavenging capacity of DPPH free radical for *C. incanus* dry herb was noted at the level of 8.04 mg d.w./mL of EC_50_ in ME extracts and of 2.6 mg d.w./mL in PBSE extracts. Figure 1 shows the distribution of the activity of compounds contained in ME and PBSE extracts of pasta supplemented with *C. incanus* leaves. Higher values of these indicators mean lower antioxidant activity. The lowest scavenging capacity of the free radical DPPH was noted in wheat pasta samples with value of 96.54 mg d.w./mL of EC_50_ in ME extracts. The highest capacity, for both used extractants, was evaluated in extracts of pasta enriched with 5% of *Cistus* leaves and it was over double more active than in samples without additive. The remaining EC_50_ values in ME ranged from 41.08 mg d.w./mL (5% of additive) to 87.21 mg d.w./mL (1% of additive). The largest differences in the increase in antioxidant activity have been noted between samples supplemented with 2 and 3% of *C. incanus* leaves. The ability to scavenge the free DPPH radicals in the tested PBSE, expressed as EC_50_ index, ranged from 75.00 to 316.22 mg d.w./mL. The scavenging activity of pasta PBSE increased with the addition of dried *Cistus* leaves amount, the most significant increase of 40% was observed between the samples with 3 and 4% of additive applied. Highly positive correlations (0.98) have been observed between antioxidant activity DPPH ME and DPPH PBSE (Table 2). The antioxidant activity of DPPH ME and PBSE in the same time was highly negatively correlated with the *C.incanus* amount (both − 0.98) and positively correlated with the FRAP PBSE (0.93 and 0.90, respectively) as well as with aroma of dry pasta (0.94 and 0.92, respectively). Marcinčák et al. (2008) found that the highest antioxidant capacity using the synthetic DPPH radical was characterized by methanol extracts of oregano (95.20%) and lemon balm (91.20%). Moreover, Chakraborty et al. (2016) reported that pasta enriched with a mix of leaves (coriander, curry, fenugreek) characterized greater ability to scavenge free radicals than extruded snacks, so supplementation of cold-pressed pasta could be an efficient way to improve nutritional quality according to the antioxidant activity of final products. But it was also found a small effect of HTST extrusion-cooking treatment on antioxidant activity of extrudates supplemented with chamomile, echinacea or elderberry fruits or flowers (Oniszczuk et al. 2015b, c, 2016a, b, respectively).

The results of the antiradical activity of free radicals against ABTS of compounds contained in ME and PBSE extracts, shown in Fig. 1, confirmed previous tendencies observed for DPPH. The antiradical activity of *C. incanus* dry herb against ABTS was characterized in ME and PBES extracts with the EC_50_ at 14.2 and 10.8 mg d.w./mL, respectively. The lowest antiradical activity was characterized by extracts of wheat pasta made without the addition of a plant with a EC_50_ at 214.73 and 59.65 mg d.w./mL in methanol and buffer extracts, respectively. Enrichment of pasta with 1% addition of dried *Cistus* leaves resulted in a 62% increase in activity against ABTS in ME and 32% in PBSE. The antiradical activity increased by over 87% in ME and 52% in PBSE when tested material contained 5% of additive. The Table 2 shows that between antioxidant activity of ABTS ME and ABTS PBSE was observed highly positive correlation (0.98). The antioxidant activity of ABTS ME and PBSE were highly positively correlated with the FRAP ME (0.98 and 0.99, respectively) and FRAP PBSE (0.93 and 0.96, respectively). However, the only antioxidant activity of ABTS PBSE was highly negatively correlated with the cooking weight index (− 0.91). The results obtained by Sant’Anna et al. (2014) suggest that the scavenging capability of the ABTS free radicals of pasta supplemented with grape marc powder is related to the presence of anthocyanins in the final product. While Sęczyk et al. (2016) observed that for wheat pasta fortified with carob flour (1–5%) inreased antiradical activity from about twofolds to 18-folds.

The results of ferric-reducing power of ME and PBSE of tested pasta are presented in Fig. 1. The increase in reduction capacity against FRAP was related to the increasing amount of *C. incanus* in the composition (Table 2). ME of wheat pasta characterized the reduced capacity against FRAP expressed as EC_50_ (71.80 mg d.w./mL). For ME and PBSE extracts of dry *C. incanus* herb the reduction capacity of FRAP was noted at 0.62 mg d.w./mL and 0.42 mg d.w./mL, respecively. The best ability to reduce ferric ions by the compounds contained in the ME of enriched pasta was found in samples with a 5% of plant leaves, it has reached the EC_50_ value of 14.32 mg d.w./mL. According Biernacka et al. (2017) the ferric-reducing power is increasing with increased amount of carob fiber in the wheat pasta. The FRAP abilities to reduce the compounds contained in the PBSE of the tested pasta were found in a range of EC_50_ from 72.95 mg d.w./mL to wheat pasta up to 25.65 mg d.w./mL for pasta with 5% of additive. So, the increase in ferric-reducing power was significant (20% in ME and 35% in PBES). However, not dependently the extraction procedure, the highest reducing capacity was observed in the pasta with the largest amount of *C. incanus* in the recipe. Nicoletti et al. (2015) reported the results of antioxidant activity against DPPH for extracts from various species of Cistus plant. They found significant differences between various types of tested plants, both as DPPH antiradical activity as well as reducing power EC_50_, in a range of 3–28 µg/mL and 142–28 µg/mL, respectively. Loizzo et al. (2013) tested antioxidant properties as radical scavenging ability and ferric reducing ability of five species of Cistus essential oils with DPPH, ABTS and FRAP tests, respectively. They found DPPH antiradical activity ranged from 499.9 to 991.9 µg/mL, ABTS ranged from 272.5 to 395.1 µg/mL and FRAP ability ranged from 0.4 to 19.4 µM Fe/g. Highly positive correlations were observed between reduction capacity against FRAP of ME and PBSE extracts and antioxidant activity measured with other methods (Table 2). Additionally, highly negative correlations were noted between the additive amount and reduction capacity against FRAP ME and PBSE (− 0.90 and − 0.94, respectively). Significant correlations have been found also with color determinants of cooked pasta as well as with sensory properties of dry products. Wheat pasta fortified with carob flour by supplementation of 1–5% had an effect on increased ability to reduce Fe_3_^+^ ions from about 100–300% (Sęczyk et al. 2016). Cárdenas-Hernández et al. (2016) noted that pasta exhibited higher phenols content than pure pasta and reduced antioxidant power once enriched with amaranth addition, especially the leaves.

### Cooking quality of pasta

Results of cooking quality of pasta enriched with *C. incanus* dried leaves are presented in Table 3. The optimum cooking time (OCT) evaluated for wheat pasta and products enriched with *C. incanus* dried leaves was 12.5 min and differences between tested samples were not significantly different (± 0.5 min), probably due to the small amount of additive used in the experiment. The cooking weight index (CWI) was found on the level of 3.28 for the wheat pasta without additive. Application of *C. incanus* dried leaves as an additive influenced variously on the cooking weight of supplemented pasta. Obtained values ranged from 3.20 when tested pasta with 1% of *C. incanus* addition to 3.36 for pasta supplemented with 3% of dried leaves added. Higher amount of additive lowered CWI because of disruption of continuous gluten matrix by the addition of herbal component with high fiber level. Nevertheless, observed differences in CWI were not significantly different. In the case of wheat pasta, fortification with carrot and oregano leaves also had no significant effect on the technological quality of the final product (Boroski et al. 2011). Cooking weight is more dependent on the preparation time as reported by Dziki and Laskowski (2005) during evaluation of spaghetti-type pasta produced from semolina, they observed the cooking weight index increased significantly from 2.7 for OCT to 3.3 during overcooking. Biernacka et al. (2018) reported the weight increase index of pasta from 2.62 to 3.00 if various wheat pasta were tested, whereas the cooking loss varied from 4.76 to 6.55%. They found significant and negative correlation was found between protein content and CL. In our study cooking loss of pasta supplemented with *C. incanus* not exceed 10% what is indicating pasta with high quality. Significant positive correlation of CWI was observed with hardness of cooked pasta (0.72, Table 2). Moreover, CL was significantly positively correlated with optimum cookin time and with taste of cooked pasta (0.69 and 0.63, respectively). As shown in Table 3 increased amount of additive in the recipe resulted in higher cooking losses from 5.7% for control wheat pasta up to 9.8% for products supplemented with 5% of *Cistus* herb. Addition of dry herbs lowered the total amount of proteins and disturbed the continuous gluten matrix by the addition of high-fiber dry plant fractions. It is well known that lower protein content results in a weaker protein matrix and higher CL so by increased addition of *Cistus* leaves the total protein content decreased (Biernacka et al. 2018). Boroski et al. (2011) found the highest soluble losses during cooking for pasta supplemented with 20% of oregano and carrot leaf meal what may be related to structural changes in the gluten chain caused by proteins in the leaf meal.

**Table 3:** Results of cooking characteristics and texture of dry and cooked wheat pasta depend on *Cistus incanus* participation

Addition of C*istus incanus* (%)	CWI (−)	OCT (min)	CL (%)	Hardness (N)	
				Dry pasta	Cooked pasta
0	3.29 ± 0.01^b^	12.5 ± 0.5^a^	5.7 ± 0.002^b^	4.68 ± 2.89^a^	0.26 ± 0.04^a^
1	4.36 ± 0.13^a^	12.0 ± 0.5^a^	6.8 ± 0.001^ab^	4.81 ± 1.15^a^	0.29 ± 0.04^ab^
2	4.12 ± 0.11^a^	14.5 ± 0.5^a^	6.7 ± 0.001^ab^	5.00 ± 1.69^a^	0.38 ± 0.10^ab^
3	4.33 ± 0.04^a^	14.5 ± 0.5^a^	8.1 ± 0.003^a^	5.16 ± 1.92^a^	0.34 ± 0.07^ab^
4	4.19 ± 0.13^a^	12.0 ± 0.1^a^	7.8 ± 0.001^a^	4.33 ± 1.62^a^	0.36 ± 0.12^ab^
5	4.56 ± 0.09^a^	12.0 ± .01^a^	9.8 ± 0.005^c^	2.79 ± 1.16^b^	0.41 ± 0.08^ab^

n = 3; mean ± SD

*CWI* cooking weight index, *OCT* optimum cooking time, *CL* cooking loss

^a–c^Means followed by the same letter within a column indicate no significant difference (*p* < 0.05) in Tukey test

Hardness of pasta was tested for dry and cooked products. Pasta cutting force as an indirect indicator of pasta hardness is a frequently determined for single or multiple pasta treads (Bouasla et al. 2016; Gull et al. 2015). Dry pasta hardness, expressed as cutting force, did not differ significantly, except of sample with the highest amount of *C. incanus* dry leaves what showed low cutting force (Table 3) as the result of weak inside structure of dry pasta and tendency to crush very easily. Hardness of cooked pasta ranged from 0.26 N for control wheat pasta up to 0.41 N for samples with 5% of dry *Cistus* added. High content of fiber in pasta recipe caused higher hardness after OTC. This could be the result of the competition for the water molecules between starch, proteins and fiber and different hydration levels of the constituents, which in turn can affect the strength of gluten network formed (La Gatta et al. 2017). Biernacka et al. (2018) found cooked pasta hardness ranged from 0.52 to 1.65 N for various types of commercial wheat pasta products based on common and durum wheat flour. Table 2 shows that hardness of cooked pasta was significantly negatively correlated with only sensory color of dry pasta (− 0.66).

### Color determination of pasta products

According to Italians, who are the leaders in Europe in the consumption of pasta, good quality pasta should have the yellow color which is desirable by consumers (Piwińska et al. 2016). Moreover, color of the product is the first evaluated parameter affecting on buying decisions. In the case of tested pasta fortified with *C. incanus* evaluation of color discriminants for dry and cooked pasta occurred with the coordinate system L*, a* and b* (Table 4). The highest brightness (value L* = 74.58) was observed for dry pasta without additives. For dry pasta the lowest value of this parameter 61.06 was noted for products fortified with 5% of *C. incanus* in the composition. There was observed significant differences between samples, especially comparing to control sample. Addition of 1–3% of dried *Cistus* leaves did not affected significantly on dry pasta brightness. While determining the brightness of cooked pasta the highest value of the L* coordinate was noted in wheat pasta without the addition of *C. incanus* with the value of 52.22. In relation to pasta before cooking the decrease in brightness occurred by approx. 30%. The lowest value of L* coordinate for cooked pasta was determined for pasta with 5% addition of dried *Cistus* leaves (40.02). Increasing the amount of *Cistus* additive caused significant reduction of cooked pasta brightness because of the brown–red shade of dry herb. There was also observed significant decrease in supplemented products brightness after cooking compared to dry pasta. The decrease in brightness of pasta could be the result of non-enzymatic browning, Maillard reactions occurred at very high temperature of drying or additives applied (Piwińska et al. 2016). Significant positive correlations (Table 2) were observed between value L* of dry pasta and value L* of cooked pasta (0.64). Furthermore, value L* of dry pasta was significantly negatively correlated with a* and b* value of dry pasta (− 0.72 and − 0.67, respectively). Also, the L* value of cooked pasta was significantly negatively correlated with additive amount, total phenolic content and b* of dry pasta whereas the positive correlations have been found between lightness of cooked pasta and its antioxidant activity (Table 2). Quite opposite tendencies were observed for b* of cooked pasta. The chromatic coordinate a*, indicating redness-greenness balance reached in-plus values what is associated with the slight brown–red tint of dry pasta. The highest a* value was determined for dry and cooked pasta with addition of 1% of *C. incanus* and was respectively 3.33 and 4.38 (Table 4). In dry pasta a small increase of redness intensity was observed according to increased level of dry *Cistus* leaves because of its brown–red shade after drying. Not significant differences were observed in a* coordinate values for cooked pasta, slide increase in redness was noted for samples with increased dried herb addition. Small differences in pasta redness before and after cooking showed similar red tint of both dry and cooked pasta. According to Biernacka et al. (2017) the effect of the color characteristic depends on the raw materials, processing parameters and especially drying conditions. The authors observed that addition of carob fiber to common wheat pasta decreased the value of the a* coordinate from 17.3 to 2.7. Uncooked pasta with spinach showed intensive green color as seen by negative a* value that indicates green shade opposite to carrot, beetroot and tomato supplemented pasta (Rekha et al. 2013). They reported the color of cooked samples was slightly lesser as compared to dry ones and leaching of color components during pasta cooking was negligible probably due to solubility in water carotenes and chlorophylls from vegetables. Significant positive correlation (0.63) was observed between value a* and b* of dry pasta. Color coordinate a* was positively correlated with additive amount and TPC whearas negative correlations have been found with antioxidant activity, especially by DPPH and FRAP radicals scavenging power (Table 5).

**Table 4:** Mean values of color coordinates L*, a* and b* of pasta depend on *Cistus incanus* participation

Addition of *Cistus incanus* (%)	Dry pasta			Cooked pasta		
	L*	a*	b*	L*	a*	b*
0	74.58 ± 2.79^c^	2.54 ± 0.82^a^	10.52 ± 0.34^d^	52.22 ± 5.45^c^	1.08 ± 0.97^b^	6.66 ± 0.35^c^
1	69.24 ± 1.47^bc^	3.33 ± 0.46^a^	14.83 ± 0.48^a^	46.08 ± 2.01^b^	4.38 ± 0.71^a^	11.90 ± 1.52^a^
2	68.45 ± 1.67^abc^	3.60 ± 0.20^ab^	15.32 ± 1.16^ab^	44.00 ± 1.84^ab^	3.40 ± 1.09^a^	12.84 ± 1.01^ab^
3	68.12 ± 1.33^abc^	4.00 ± 1.27^ab^	16.05 ± 0.21^abc^	44.75 ± 2.90^ab^	4.08 ± 0.89^a^	13.88 ± 1.72^ab^
4	62.12 ± 4.33^ab^	3.76 ± 0.56^ab^	16.68 ± 0.63^bc^	43.36 ± 0.70^ab^	4.75 ± 0.21^a^	14.43 ± 0.29^ab^
5	61.06 ± 5.03^a^	5.12 ± 0.45^b^	17.66 ± 1.14^c^	40.02 ± 3.27^a^	5.24 ± 1.52^a^	14.95 ± 1.13^b^

n = 5; mean ± SD

L* brightness, a* redness(+)–greenness(−) balance, b* yellowness(+)–blueness(−) balance

^a–d^Means followed by the same letter within a column indicate no significant difference (*p* < 0.05) in Tukey test

**Table 5:** Results of acceptability of dry and cooked wheat pasta supplemented with addition of dried *Cistus incanus* leaves

Addition of *Cistus incanus* (%)	Dry pasta				Cooked pasta					
	Appearance	Color	Aroma	Overall acceptability	Appearance	Color	Aroma	Taste	Texture	Overall acceptability
0	8.00 ± 0.63^b^	7.40 ± 0.49^b^	7.24 ± 0.48^b^	7.55	6.67 ± 0.82^ab^	5.71 ± 0.98^a^	7.04 ± 0.63^a^	7.07 ± 0.85^ab^	7.67 ± 0.67^b^	6.83
1	8.07 ± 0.68^b^	7.20 ± 0.75^b^	7.13 ± 0.72^b^	7.47	7.91 ± 0.75^b^	7.28 ± 0.65^b^	6.37 ± 0.90^a^	7.44 ± 0.91^b^	8.28 ± 0.78^b^	7.46
2	7.93 ± 0.57^b^	7.11 ± 0.92^b^	7.04 ± 0.92^b^	7.36	7.44 ± 0.72^b^	6.67 ± 1.03^ab^	6.13 ± 1.22^a^	7.00 ± 0.92^ab^	7.55 ± 0.88^b^	6.96
3	6.27 ± 0.85^ab^	4.24 ± 0.97^a^	5.13 ± 0.98^a^	5.21	5.33 ± 0.87^ab^	5.71 ± 0.93^a^	6.22 ± 1.01^a^	6.53 ± 1.05^ab^	7.27 ± 1.16^b^	6.21
4	4.40 ± 0.95^a^	4.09 ± 0.89^a^	5.02 ± 1.13^a^	4.50	5.29 ± 0.96^ab^	5.53 ± 1.11^a^	6.15 ± 1.89^b^	6.40 ± 1.20^ab^	6.33 ± 1.21^b^	5.94
5	4.07 ± 0.85^a^	3.95 ± 0.84^a^	4.88 ± 0.79^a^	4.30	2.95 ± 0.87^a^	5.06 ± 0.83^a^	5.58 ± 0.61^a^	5.53 ± 0.91^a^	3.31 ± 1.13^a^	4.48

^a–d^Means followed by the same letter within a column indicate no significant difference (*p* < 0.05) in Tukey test

During the assessment of the b* coordinate (Table 4), the highest intensity of the yellow shade was determined for dry pasta with 5% *C. incanus* content (17.66), while the smallest value, 10.52, was determined during testing wheat pasta without the addition of *C. incanus*. For cooked pasta, the b* coordinate value assumed a maximum value of 14.95 during evaluating the color of the pasta with a 5% *C. incanus*, while the lowest intensity of yellowness 6.66 was characterized by wheat pasta as control sample. The increase in b* values for dry pasta was 67% according to color of control sample, while after cooking increase in yellowness was more significant (124%). Difference in yellowness between dry and cooked products was about 16–20% for pasta with the same content of *C. incanus*, but for control pasta difference was more visible (37%). Reduction in yellow color intensity of cooked pasta could be due to swelling of pasta and conversion of pigments resulting in decrease in yellowness during cooking, but all the tested pastas had good attractive color after cooking. b* value of dry pasta was positively correlated with amount of additive, TPC, a* and value b* of cooked pasta but negatively correlated with antioxidant activity for all the tested extracts, with L* of cooked pasta as well as with sensory characteristics of dry products (Table 2).

### Sensory evaluation of pasta

During the sensory evaluation of dry pasta (Table 5) the highest scores of the overall acceptability were noted for the control pasta and samples enriched with 1 and 2% of *C. incanus* added. With the increase of the amount of herbaceous additive, lower notes were observed for the color assessment, because of higher additive level visually resulted in the loss of the color uniformity. The increasing amount of the additive in the evaluation of raw pasta resulted in lowering the ratings for the external appearance. Most likely, this was due to the appearance of dark spots that resulted from the addition of *C. incanus*. The increase of *C. incanus* addition in pasta recipe also adversely affected the assessment of the aroma because of too intensive herbal flavor resulted in lowering the scores for the aroma of pasta with an additive content above 3%. The overall quality counted as mean of all assessed features showed that the control pasta and products with 1 and 2% of the additive content received the score 7.5, which constituted of 83.3% of the possible points. Pasta with 3% *C. incanus* received approximately about 30% less points than the top ones. The pasta with the addition of 5% of the herb supplement was rated the worst, receiving 4.3 points, which constituted 47.7% of total points to be awarded. Table 2 shows that appearance, color and aroma of dry pasta as well as appearance of cooked pasta were negatively correlated with additive amount. Assessment of cooked pasta sensory attributes showed the highest overall acceptability was found for pasta supplemented with 1% of *C. incanus* dry leaves. The pasta made with this recipe after cooking characterized the best appearance, color, taste and texture. When assessing the aroma, as compared to pasta with 1% of the additive, slightly higher scores were noted for pasta with 4% of *C. incanus* content. The lowest rated product was cooked pasta with 5% content of additive, the lowest scores for all sensory attributes were noted for this product. Significant positive correlation (0.65) was observed between the taste and texture of cooked pasta (Table 2). Whereas, as reported by La Gatta et al. (2017), supplementation of pasta with high-fiber bran fractions may cause a weakening of the gluten protein network and may have a detrimental effect on its cooking and sensory quality. So the level of fibrous additives for pasta supplementation must combine nutritional properties, proper cooking quality and sensory attractiveness. Supplementation of the health ingredients should have no effect on the palatability as well as the consumer preference. Apart from the additional health benefits it offers, it should be rather delicious as well (Krishnan and Prabhasankar 2012). Taking all these into account it can be stated that pasta with maximum level of 3% of *C. incanus* addition is still acceptable as well as presents improved nutritional characteristics.

## Conclusion

Enrichment of common wheat pasta with the addition of *C. incanus* resulted in increasing the total phenolics content and also contributed in increasing the antioxidant activity of supplemented pasta. The content of total phenolic compounds found in enriched pasta extracted with BE showed better extractability of these components from dry pasta than in ME. The highest radical scavenging capacity against DPPH, for both used extractants, was evaluated in extracts of pasta enriched with 5% of *Cistus* leaves and over double more activity was noted than in samples without additives. The best ability to reduce ferric ions by the compounds contained in the ME of enriched pasta was found for samples with a 5% of plant leaves. The introduction of *C. incanus* to the pasta dough composition resulted in a slight increase in the cooking weight of pasta. The highest value of this indicator was obtained when testing pasta with 3% of additive. Increased addition of dry *Cistus* herb caused higher cooking loss and increased hardness of cooked pasta. The increasing addition of *C. incanus* had insignificant effect on the a* color coordinate of supplemented pasta, the only slight decrease of brightness and significant increase of yellowness were observed if the amount of additive raised. After cooking supplemented pasta was more darker and less yellow in color. On the base of the sensory evaluation it is recommended fortification of common wheat pasta with maximum 3% of *C. incanus* due to overall acceptability notes. This additive due to its high phenolics content and antioxidant activity as well as proper quality characteristics have a strong potential to be implemented in practice to produce supplemented pasta. It would be interesting for Authors to find more applications for *C. incanus* herb in food industry and some research will be continued in this field.
